# Fluoroquinolone Use and *Clostridium difficile*–Associated Diarrhea

**DOI:** 10.3201/eid0906.020385

**Published:** 2003-06

**Authors:** Margaret E. McCusker, Anthony D. Harris, Eli Perencevich, Mary-Claire Roghmann

**Affiliations:** * University of Maryland, Baltimore, Maryland, USA; † Veterans Affairs Maryland Health Care System, Baltimore, Maryland, USA

**Keywords:** Antibiotics, case-control study, *Clostridium difficile*, diarrhea, fluoroquinolones, nosocomial infections, dispatch

## Abstract

We performed a case-control study to evaluate the association between antibiotic use and *Clostridium difficile*–associated diarrhea (CDAD), matching for admission unit and time at risk for CDAD. A multivariable regression model showed that treatment with fluoroquinolones (odds ratio 12.7; 95% confidence interval 2.6 to 61.6) was the strongest risk factor for CDAD.

*Clostridium difficile*–associated diarrhea (CDAD) is a leading cause of nosocomial diarrhea in the United States (*1*–*4*). On average, compared with patients without this disease, patients in whom CDAD is diagnosed have hospital stays that are 3.6 days longer and additional hospital costs of $3,699 (*5*). Research has shown that patients are usually exposed to *C. difficile* throughout their hospitalizations and that antibiotic use promotes the acquisition of this organism (*1*). The outcome of acquisition, which may be colonization or infection with *C. difficile,* is thought to be determined primarily by patient factors including advanced age and severity of underlying illness, which may compromise the ability to mount an immune response against the bacteria (*6*).

Clindamycin, penicillins, and cephalosporins have been associated with CDAD (*4*). Although fluoroquinolones are not currently believed to cause this illness, several case reports of fluoroquinolone-associated *C. difficile* diarrhea have been published (*6*–*12*). A case-control study of patients at an acute-care hospital identified ciprofloxacin use as a strong risk factor for nosocomial CDAD (*13*). The broadened anti-anaerobic spectrum of newer fluoroquinolones raises the issue of whether therapy with these agents can predispose this illness to develop in patients (*14*).

Increasing rates of *C. difficile* infection in cases dispersed throughout our healthcare system prompted an examination of patient-associated risk factors for CDAD. We hypothesized that patients in whom CDAD was diagnosed were more likely to have received antibiotics of which use had increased over the past year and that differences in antimicrobial drug–prescribing patterns could account for the observed increase in cases.

## The Study

The Veterans Affairs Maryland Health Care System (VAMHCS) provides all medical services from intensive care to ambulatory and pharmacy services for approximately 36,000 veterans at four separate inpatient sites. A total of 778 beds are available for inpatient care, 120 of which are dedicated to acute medical and surgical care. Cases were defined as patients who were admitted to a VAMHCS institution from January 1, 2001, to June 30, 2001, who had a new onset of diarrhea documented in their medical records at least 72 hours after admission, a subsequent positive *C. difficile* toxin A enzyme immunoassay result (Wampole Laboratories, Cranbury, NJ), and no known history of CDAD. Patients with other reasons for diarrhea, such as laxative use, were excluded. The date of the positive *C. difficile* toxin test was considered to be the date of CDAD diagnosis.

We selected two controls per case from patients admitted to the VAMHCS institution for at least 48 hours during the same 6-month period as the case-patients. Controls were matched to the case-patients by unit of admission and length of time at risk for development of CDAD (defined below). We attempted to find two controls for each case with a time at risk within 5 days of that of the case. When finding such a control was not possible, we selected a control with the next closest length of time at risk. Controls had no known history of CDAD and did not receive oral metronidazole during their hospital stay in order to minimize misclassification of controls that might be cases.

We collected data by reviewing electronic medical records. Since antibiotic use up to 8 weeks before the CDAD diagnosis has been implicated as causing infection in previous studies (*1*,*3*), we examined both inpatient and outpatient antibiotic use within the 6 weeks before diagnosis of CDAD for cases and for 6 weeks before hospital discharge for controls. Specific use of clindamycin, cephalosporins, fluoroquinolones, piperacillin-tazobactam, and any other antibiotic drugs was recorded. The number of days that fluoroquinolones were administered to each patient was determined from medication orders and nursing notes. Length of time at risk for CDAD was defined as the number of days from admission to development of the illness for cases and the number of days from admission to discharge for controls. Demographic variables and details of hospital admission were also recorded, including the unit where CDAD was diagnosed (cases) or the admission unit (controls).

We compared characteristics of cases and controls with the Wilcoxon rank-sum test for continuous variables and the Fisher exact test for categorical variables. Matched analysis of the association between individual variables and case or control status was performed by using Cochran-Mantel-Haenszel estimates. Conditional logistic regression was used to assess the odds of CDAD developing in a patient. Variables significantly associated with CDAD in our preliminary analysis were included in the multivariable regression model. Confounding was assessed by checking for a >10% change in the coefficient estimate of covariates between models. A p value <0.05 was considered significant; all statistical tests were two-tailed. Statistical analyses were performed by using SAS software version 8.1 (SAS Institute, Inc., Cary, NC).

Thirty patients met the case definition during the study period; 60 controls were selected. The mean age of cases and controls was not significantly different (Table 1). With the exception of one female control, all patients were male. Despite matching, case-patients had a longer length of time at risk for CDAD, but the difference between cases and controls was not statistically significant (p=0.18). Of both cases and controls, 20% were admitted to general medical units, 23% to general surgical units, 27% to subacute or long-term care, 17% to the medical intensive-care unit, and 13% to the surgical intensive-care unit.

**Table 1:** Characteristics of CDAD cases and matched controls, Veterans Affairs Maryland Health Care System, January 1, 2001–June 30, 2001

Characteristic	Cases (n=30)^a^	Controls (n=60)^a^	p value
Age, median y	72 (66–79)^b^	67 (56–76)^b^	0.30
Diagnosis causing admission			
Infectious	11 (37)	11 (18)	0.07
Cardiovascular	5 (17)	14 (23)	0.59
Neurologic/psychiatric	3 (10)	15 (25)	0.16
Gastroenterologic	2 (7)	4 (7)	1.00
Respiratory	1 (3)	5 (8)	0.66
Other	8 (27)	11 (18)	0.42
Antibiotics within 6 weeks	30 (100)	38 (63)	<0.01
Cephalosporins	7 (23)	20 (33)	0.30
Clindamycin	9 (30)	7 (12)	0.03
Fluoroquinolones	22 (73)	15 (25)	<0.01
Piperacillin/tazobactam	12 (40)	18 (30)	0.30
All other antibiotics	17 (57)	27 (45)	0.30
Days at risk for CDAD^c^, median	21(10–30)^b^	13(7–25)^a^	0.18

^a^Unless otherwise indicated, values in parentheses show percentages. ^b^Value in parentheses shows interquartile range. ^c^CDAD, *Clostridium difficile*–associated diarrhea.

All 30 (100%) case-patients received antibiotics during the 6 weeks before their CDAD diagnosis. In the comparable 6-week window, 38 (63%) of the controls received antibiotics (p<0.01 for difference). Both clindamycin and fluoroquinolones were administered to a significantly higher proportion of cases than controls. For the patients who received fluoroquinolones, levofloxacin was prescribed most often for both cases (60%) and controls (60%), followed by ciprofloxacin (45% and 27%, respectively), and gatifloxacin (14% and 20%, respectively). These differences were not statistically significant. Among the patients who received fluoroquinolones, 41% of case-patients and 27% of controls received >1 week of fluoroquinolones (p=0.01).

Matched univariate analysis of risk factors for CDAD showed that fluoroquinolone use (odds ratio [OR] 13.5; 95% confidence intervals [CI] 3.1 to 58.8) and clindamycin use (OR 3.1; 95% CI 1.0 to 9.4) were associated with developing this illness (Table 2). The results of the multivariable analysis are shown in Table 3. After confounding from other antimicrobial agents was controlled for, fluoroquinolone use was significantly associated with an increased risk of developing CDAD (OR 12.7; 95% CI 2.6 to 61.6).

**Table 2:** Matched univariate analysis of risk factors for *Clostridium difficile–*associated diarrhea

Risk factor	Odds ratio	95% confidence interval	p value
Fluoroquinolone	13.5	3.1 to 58.8	<0.01
Clindamycin	3.1	1.0 to 9.4	0.05
Piperacillin/tazobactam	1.9	0.7 to 5.1	0.24
Cephalosporins	0.6	0.2 to 1.7	0.32
All other antibiotic drugs	1.6	0.7 to 4.1	0.28

**Table 3:** Multivariable model of risk factors for Clostridium *difficile*–associated diarrhea in cases (n=30) versus controls (n=60), controlling for days at risk

Risk factor	Odds ratio	95% confidence interval
Fluoroquinolones	12.7	2.6 to 61.6
Cephalosporins	0.4	0.1 to 1.5
Clindamycin	2.2	0.5 to 9.1

## Conclusions

Although ciprofloxacin-induced CDAD has been reported, early reports were dismissed as being due to other causes of diarrhea, including infection with *Salmonella* and previous treatment with a different antibiotic (*6*–*8*,*12*). A group of bone marrow transplant patients who received ciprofloxacin monotherapy for prophylaxis against infection had no instances of CDAD, but two concurrent reports included cases of CDAD associated exclusively with ciprofloxacin (*9*,*10*,*15*). Another report implicated levofloxacin in eight of nine cases of CDAD in a nursing home (*11*). Fluoroquinolone use was also identified as an independent predictor of a positive *C. difficile* toxin assay in hospitalized patients (*16*). In addition, a case-control study of patients at an acute-care hospital identified ciprofloxacin use as a strong risk factor for nosocomial CDAD with an OR >5 in each regression model (*13*). Thus, our study is consistent with more recent reports that implicate fluoroquinolone use as a risk factor for CDAD.

We found that the association between fluoroquinolones and CDAD is stronger than the association between clindamycin and CDAD. However, the confidence intervals are wide because of the small sample size and overlap for the estimates, making a conclusion that fluoroquinolones are a stronger risk factor for CDAD than clindamycin inappropriate from our study. Because patients commonly receive more than one antibiotic, accurately measuring the effects of individual antibiotics in an observational study is difficult. Concurrent prescribing of clindamycin and fluoroquinolones may have biased the estimates of the OR; however, only 32% of patients who received fluoroquinolones also received clindamycin. In the case-control study of acute-care patients by Yip et al., ciprofloxacin was also a stronger risk factor than clindamycin (*13*).

Our study has a number of limitations. Since the study was retrospective and we did not perform surveillance cultures for *C. difficile,* we could not ascertain when this organism was acquired; however, all cases received antibiotics before the diagnosis of CDAD. Thus, we conclude that fluoroquinolones are clearly associated with *C. difficile* infection. On the basis of our study design, we could not determine whether fluoroquinolones increase acquisition or promote infection once *C. difficile* is acquired. Although we did not specifically assess the role of patient-to-patient transmission in this study, we selected case-patients and controls from the same hospital units and with a similar risk period for developing the illness. Given the strength of the association between fluoroquinolone use and CDAD, more precise controlling for patient-to-patient transmission is unlikely to eliminate the association.

If fluoroquinolone use is a stronger contributing factor to *C.* *difficile* infection than other antibiotics, then restriction of fluoroquinolone use among inpatients would result in decreased CDAD rates. Climo et al. reported a decrease in the incidence of CDAD at their institution after implementing a formulary restriction of clindamycin (*17*). However, the decision to restrict fluoroquinolone use would need to be weighed against the clinical advantages of using fluoroquinolones, such as convenient dosing and excellent oral bioavailability (i.e., the ability of a drug to achieve high serum levels when taken by mouth). We observed a strong association between fluoroquinolone use and CDAD in both our acute-care and long-term–care patients, which supports a number of reports implicating fluoroquinolones in the development of CDAD (*6*–*11*,*13*). A prospective study of this association is warranted, given the increasing use of fluoroquinolones and the excess complications and costs associated with *C. difficile* infection (*5*).
